# Distribution of *Ecnomus* McLachlan, 1864 (Trichoptera, Ecnomidae) from the Lower Mekong River with a description of *Ecnomusstungtrengensis* new species

**DOI:** 10.3897/BDJ.10.e94936

**Published:** 2022-12-01

**Authors:** Pongsak Laudee, Hans Malicky, Kriengkrai Seetapan, Penkhae Thamsaenanupap, Supawit Karnkasem, Chanda Vongsombath, Sai Sein Lin Oo, Chamroeun Kong, Pome Zalyan, John C. Morse

**Affiliations:** 1 Faculty of Innovative Agriculture and Fishery Establishment Project, Prince of Songkla University, Surat Thani Campus, Surat Thani, Thailand Faculty of Innovative Agriculture and Fishery Establishment Project, Prince of Songkla University, Surat Thani Campus Surat Thani Thailand; 2 Sonnengasse 13, Lunz am See, Austria Sonnengasse 13 Lunz am See Austria; 3 School of Agriculture and Natural Resources, University of Phayao, Phayao, Thailand School of Agriculture and Natural Resources, University of Phayao Phayao Thailand; 4 Faculty of Environment and Resource Studies, Mahasarakham University, Maha Sarakham, Thailand Faculty of Environment and Resource Studies, Mahasarakham University Maha Sarakham Thailand; 5 Faculty of Environmental Sciences, National University of Laos, Dong Dok Campus, Vientiane, Laos Faculty of Environmental Sciences, National University of Laos, Dong Dok Campus Vientiane Laos; 6 No.208, Southern Quarter, Muse Township, Shan State, Myanmar No.208, Southern Quarter Muse Township, Shan State Myanmar; 7 Kampong Speu Institute of Technology, Kampong Speu, Cambodia Kampong Speu Institute of Technology Kampong Speu Cambodia; 8 Department of Zoology, University of Kalay, Myanmar, Kalay, Myanmar Department of Zoology, University of Kalay, Myanmar Kalay Myanmar; 9 Clemson University, Clemson, United States of America Clemson University Clemson United States of America

**Keywords:** caddisflies, biodiversity, aquatic insects, the Mekong River, Southeast Asia

## Abstract

**Background:**

Trichoptera were surveyed from four different physiographic regions of the Lower Mekong River, including the Northern Highlands, the Khorat Plateau, the Tonle Sap Basin and the Mekong Delta in Myanmar, Laos, Thailand, Cambodia and Vietnam.

**New information:**

Twenty-three species of the genus *Ecnomus*, including a new species, were identified and mapped. *Ecnomusmammus* Malicky & Chantaramongkol, 1993 is a common species which is found from Tonle Sab Basin and Mekong Delta. *Ecnomusalkmene* Malicky & Chantaramongkol, 1997, *E.volovicus* Malicky & Chantaramongkol, 1993 and *Ecnomusquordaio* Malicky, 1993 are the common species in the area of the Northern Highlands and the Khorat Plateau. *Ecnomusplotin* Malicky & Laudee 2010 is found in the Mekong Delta. *Ecnomustriangularis* Sun, 1997 is a new species record for Southeast Asia. In addition, *E.stungtrengensis* sp. n. is described and illustrated. The male genitalia of *E.stungtrengensis* sp. n. are clearly different from those of other species in the genus *Ecnomus* by the shape of superior appendages which are slender and bent downwards distally in lateral view.

## Introduction

Based on the number of species, *Ecnomus* is the largest of seven genera in the family Ecnomidae (Johanson and Espeland 2022, Morse 2022). This genus has been reported almost worldwide, except the Americas (Cartwright 1990, Li and Morse 1997, Malicky 2010b, Pandher and Saini 2013, Kuhara 2016). In the Oriental Region, 174 species have been reported (Malicky 2010a, Laudee and Malicky 2018, Laudee et al. 2020, Mey and Malicky 2021, Morse 2022). Larvae of *Ecnomus* spp. are reported from many kinds of aquatic habitats, including both of lentic and lotic ecosystems and rhithon or potamon habitats (Dugeon 1999). Thapanya et al. (2004) reported seven species of *Ecnomus* from Doi Inthanon and Doi Suthep-Pui National Parks, northern Thailand, where the habitat is mountain streams. Laudee and Prommi (2011) reported that the number of species of *Ecnomus* was the greatest in the Tapi River and that *E.atevalus* Malicky & Chantaramongkol, 1993, *E.paget* Malicky & Chantaramongkol, 1997 and *E.votticius* Malicky & Chantaramongkol, 1993 were found near the Tapi River mouth, southern Thailand. Moreover, two ecnomid species, *E.crepidulus* Mosely, 1932 and *E.selangor* Wells & Yule, 2008 were reported from a tropical peat swamp in Selangor, Peninsular Malaysia (Wells and Yule 2008). Mey and Malicky (2021) reported that *E.oppositus* Martynov, 1935 lives in northern Inle Lake, Shan State, Myanmar. A report of *Ecnomus* species in the Mekong River was recently published by Laudee et al. (2020), who described two new species, *E.petchanaae* Laudee & Malicky, 2020 and *E.boonsawaengae* Malicky & Vongsombath, 2020 from Mekong River tributaries in Laos, the latter species from a waterfall.

The Mekong River is the longest river in Southeast Asia. The river is divided into two parts, the Upper and Lower Mekong River. The Lower Mekong River extends from the Chinese border in Shan State, Myanmar, to the Mekong Delta in southern Vietnam (Mekong River Commission 2022). Four different physiographic regions of the Lower Mekong River, including the Northern Highlands, the Khorat Plateau, the Tonle Sap Basin and the Mekong Delta, are areas of Thailand, Laos , Cambodia and Vietnam (Mekong River Commission 2022). We surveyed the species diversity of Trichoptera from the Lower Mekong River. The distributions of species in genus *Ecnomus* spp. along the Lower Mekong River are discussed. In addition, a new species of *Ecnomus* is described and illustrated.

## Materials and methods

Adult caddisflies specimens were collected by a UV pan light trap (12 V, 10 W) near streams and rivers overnight at each site. The Trichoptera specimens were preserved in 70% ethanol and manually sorted afterwards at Prince of Songkla University, Surat Thani Laboratory. The adult male genitalia were excised and macerated by heating in 10% potassium hydroxide (KOH) at 60^o^C for 30–60 minutes. Only male caddisflies were identified and counted.

**Figure 1.**
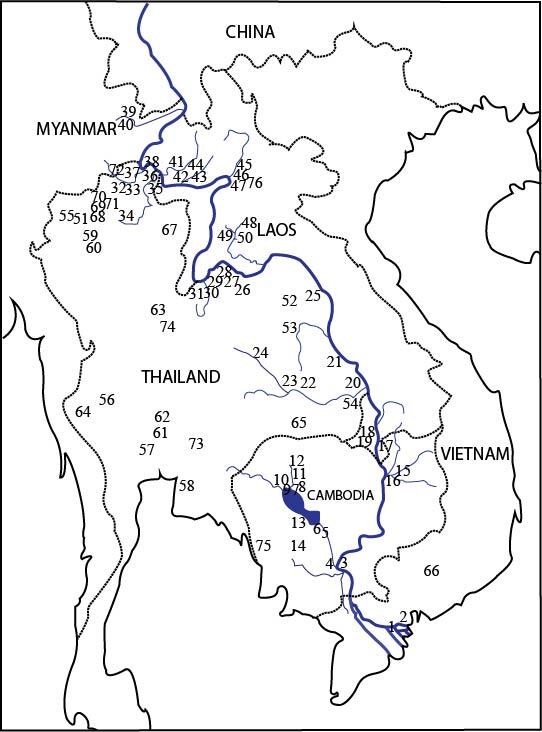
Study sites for collection of *Ecnomus* spp. from the Lower Mekong River and its tributaries,Thailand, Cambodia, Laos and Vietnam, with numbers corresponding to the details listed in the text. Outline of the map is redrawn from www.stimson.org.

The caddisflies of the genus *Ecnomus* were plotted on the Lower Mekong River map to show the distribution of the species (Fig. 1). The data of some caddisflies of the genus *Ecnomus* from Thailand, Laos, Cambodia and Vietnam previously collected by Hans Malicky were plotted to show the distribution of the species in adjacent areas.

The specimen collection sites were as follows:

1. Vietnam, Ben Tre, Khu Pho 3, Bentre Riverside Resort, Ham Luong River, 10°13’18”N 106°20’58”E, 5 December 2019, 1 m a.s.l., leg. Pongsak Laudee.
2. Vietnam, My Tho, Cu Lao Thoi Son, Mekong Taste, Mekong River, 10°20’26”N 106°21’47”E, 8 December 2019, 3 m a.s.l., leg. Pongsak Laudee.
3. Cambodia, Phnom Penh Province, Mekong River, 11°33’52”N 104°56’01”E, 10 April 2019, 8 m a.s.l., leg. Pongsak Laudee.
4. Cambodia, Phnom Penh Province, Tonle Sap River, 11°38’31”N 104°52’34”E, 10 April 2019, 13 m a.s.l., leg. Pongsak Laudee.
5. Cambodia, Kampong Chhnang Province, Tonle Sap River, 11°53’10”N 104°46’16”E, 11 April 2019, 11 m a.s.l., leg. Pongsak Laudee.
6. Cambodia, Kampong Chhnang Province, Tonle Sap River, 11°50’13”N 104°47’57”E, 11 April 2019, 11 m a.s.l., leg. Pongsak Laudee.
7. Cambodia, Siem Reap Province, Kampong Phluk, Tonle Sab, 13°11’25”N 103°58’10”E, 12 April 2019, a.s.l. 4 m a.s.l., leg. Pongsak Laudee.
8. Cambodia, Siem Reap Province, Kampong Phluk, Tonle Sab, 13°11’42”N 103°58’26”E, 12 April 2019, 5 m a.s.l., leg. Pongsak Laudee.
9. Cambodia, Siem Reap Province, Chong Khneas, Tonle Sab, 13°14’04”N 103°49’37”E, 3 May 2019, 7 m a.s.l., leg. Pongsak Laudee.
10. Cambodia, Siem Reap Province, Chong Khneas, Tonle Sab, 13°04’09”N 104°05’25”E, 3 May 2019, 9 m a.s.l., leg. Pongsak Laudee.
11. Cambodia, Siem Reap Province, Siem Reap River, 13°23’40”N 103°52’34”E, 11 April 2019, 20 m a.s.l., leg. Pongsak Laudee.
12. Cambodia, Siem Reap Province, Siem Reap River, 13°24’09”N 103°52’48”E, 6 May 2019, 22 m a.s.l., leg. Pongsak Laudee.
13. Cambodia, Pursat Province, Kampong Luong, Tonle Sap, 12°34’06”N 104°12’30”E, 7 May 2019, 7 m a.s.l., leg. Pongsak Laudee.
14. Cambodia, Kampong Speu Province, Chreav waterfall, 11°58’00”N 104°14’32”E, 8 May 2019, 353 m 353 m a.s.1., leg. Pongsak Laudee.
15. Cambodia, Stung Treng Province, Stung Treng, Tonle Sekong River, 13°32’00”N 105°58’56”E, 9 April 2019, 42 m a.s.l., Pongsak Laudee.
16. Cambodia, Stung Treng Province, Stung Treng, Mekong River, 13°47’10”N 105°94’03”E, 9 April 2019, 40 m a.s.l., Pongsak Laudee.
17. Laos, Pak Se Province, Champasak, Khonphapheng Waterfall, 13°57’30”N 105°59’14”E, 8 April 2019, 64 m a.s.l., Pongsak Laudee.
18. Laos, Pak Se Province, Champasak, Don Khon, Mekong River, 13°56’40”N 105°55’06”E, 8 April 2019, 84 m a.s.l., Pongsak Laudee.
19. Laos, Pak Se Province, Champasak, Don Khon, Mekong River, 13°56’40”N 105°55’06”E, 8 April 2019, 64 m a.s.l., Pongsak Laudee.
20. Thailand, Ubon Ratchathani Province, Khong Chiam District, Mekong River, 15°19’21”N 105°29’46”E, 10 November 2021, 100 m a.s.l., leg. Pongsak Laudee.
21. Thailand, Ubon Ratchathani Province, Sam Phan Bok, Mekong River, 15°47’42”N 105°23’46”E, 10 November 2021, 110 m a.s.l., leg. Pongsak Laudee.
22. Thailand, Ubon Ratchathani Province, Ban Thasala, Chi River, 15°16’40”N 104°38’32”E, 28 May 2019, 121 m a.s.l., leg. Penkhae Thamsenanupap.
23. Thailand, Ubon Ratchathani Province, Ban Kutkasian, Chi River, 15°32’28”N 104°54’70”E, 28 May 2019, 123 m a.s.l., leg. Penkhae Thamsenanupap.
24. Thailand, Maha Sarakham Province, Ban Tha Ngam, Chi River, 16°16’04”N 103°04’57”E, 30 May 2019, 154 m a.s.l., leg. Penkhae Thamsenanupap.
25. Thailand, Nakhon Phanom Province, Mueang Nakhon Phanom, Mekong River, 17°26’39”N 104°44’56”E, 9 November 2021, 140 m a.s.l., leg. Pongsak Laudee.
26. Thailand, Nong Khai Province, Thantip Waterfall, 18°07’26”N 102°11’07”E, 26 May 2019, 327 m a.s.l., leg. Penkhae Thamsenanupap.
27. Thailand, Nong Khai Province, Keangkong Chommork Hotel, 18°11’14”N 102°10’07”E, 190 m a.s.l., 26 May 2019, leg. Penkhae Thamsenanupap.
28. Thailand, Loei Province, Chiang Khan, Huai Hia stream, 18°00’00”N 101°45’48”E, 25 May 2019, 223 m a.s.l., leg. Penkhae Thamsenanupap.
29. Thailand, Loei Province, Kaeng Kud Ku, Mekong River, 17°54’26”N 101°42’06”E, 25 May 2019, 202 m a.s.l., leg. Penkhae Thamsenanupap.
30. Thailand, Loei Province, Chiang Khan, Mekong River, 17°51’15”N 101°36’16”E, 26 May 2019, 212 m a.s.l., Penkhae Thamsenanupap.
31. Thailand, Loei Province, Chiang Khan, Loei River, 17°51’11”N 101°36’23”E, 26 May 2019, 217 m a.s.l., Penkhae Thamsenanupap.
32. Thailand, Chiang Rai Province, Ko Pha Sing, Kok River, 19°58’34”N 99°67’33”E, 10 December 2018, 420 m a.s.l., leg. Pongsak Laudee.
33. Thailand, Chiang Rai Province, Ban Doi Hang, Kok River, 19°56’22”N 99°45’27”E, 10 December 2018, 400 m a.s.l., leg. Pongsak Laudee.
34. Thailand, Phayao Province, Mae Tam, Ing River, 19°9’46”N 99°55’43”E, 12 December 2018, 380 m a.s.l., leg. Pongsak Laudee.
35. Thailand, Phayao Province, Phu Sang, Pueai stream, 19°40’07”N 100°23’15”E, 12 December 2018, 480 m a.s.l., leg. Pongsak Laudee.
36. Thailand, Chiang Rai Province, Nong Buak Chin, Ing River, 20°7’35”N 100°23’36”E, 14 December 2018, 360 m a.s.l., Pongsak Laudee.
37. Thailand, Chiang Rai Province, Rob Wiang, Kok River, 19°55’20”N 99°52’47”E, 15 December 2018, 400 m a.s.l., Pongsak Laudee.
38. Thailand, Chiang Rai Province, Golden Triangle, Mekong River, 20°19’50”N 100°05’05”E, 16 December 2018, 400 m a.s.l., leg. Pongsak Laudee.
39. Myanmar, Shan State, Keng Tung, Mekheun River, 21°31’44”N 99°34’25”E, 11 February 2020, 720 m a.s.l., leg. Pongsak Laudee.
40. Myanmar, Shan State, Keng Tung, Mekheun River, 21°30’43”N 99°35’17”E, 11 February 2020, 690 m a.s.l., leg. Pongsak Laudee.
41. Laos, Bokeo Province, Ban Namkha, Nam Kha Stream, 20°07’32”N 100°46’53”E, 27 April 2019, 380 m a.s.l., leg. Pongsak Laudee.
42. Laos, Oudomxay Province, Ban Pak Beng, Mekong River, 19°53’12”N 101°07’44”E, 28 April 2019, 320 m a.s.l., leg. Pongsak Laudee.
43. Laos, Oudomxay Province, Ban Pak Beang, Nam Beng River, 19°54’59”N 101°09’48”E, 1 March 2019, 340 m a.s.l., leg. Pongsak Laudee.
44. Laos, Oudomxay Province, Ban Pak Beng, Namkasan Stream, 19°55’53”N 101°10’11”E, 1 March 2019, 360 m a.s.l., leg. Pongsak Laudee.
45. Laos, Luang Prabang Province, Ban Pak Ou, Nam Ou River, 19°54’59”N 102°09’48”E, 3 March 2019, 300 m a.s.l., leg. Pongsak Laudee.
46. Laos, Luang Prabang Province, EP Camp, Mekong River, 20°01’46”N 102°13’43”E, 2 March 2019, 280 m a.s.l., leg. Pongsak Laudee.
47. Laos, Luang Prabang Province, Mekong River, 19°53’53”N 102°08’01”E, 2 March 2019, 280 m a.s.l., leg. Pongsak Laudee.
48. Laos, Vientiane Province, Vang Vieng, Nam Song River, 18°55’43”N 102°26’34”E, 4 March 2019, 240 m a.s.l., Pongsak Laudee.
49. Laos, Vientiane Province, Nam Ngum, Nam Lik-Ngum River, 18°31’29”N 102°31’19”E, 6 April 2019, 180 m a.s.l., Pongsak Laudee.
50. Laos, Vientiane Province, Nam Ngum, Nam Ngum River, 18°31’29”N 102°31’37”E, 6 April 2019, 180 m a.s.l., Pongsak Laudee.
51. Thailand, Chiang Mai Province, Chom Thong District, Huai Mae Ya stream, 19°15’N 98°29’E, 530 m a.s.l., H. Malicky.
52. Thailand, Sakon Nakhon Province, Huai Yang sub-District,Phu Phan National Park, 17°04’N 103°58’E, 233 m a.s.l., H. Malicky.
53. Thailand, Sakon Nakhon Province, Ban Huai Huat, Phu Pha Yon National Park, 17°04’N 103°58’E, 199 m a.s.l., H. Malicky.
54. Thailand, Ubon Ratchathani Province, Khong Chiam District, Kaeng Tana National Park, 15°20’N 105°30’E, 101 m a.s.l., H. Malicky.
55. Thailand, Mae Hong Son Province, Pai District, Muang Pai Resort, 19°23’N 98°25’E, 607 m a.s.l., H. Malicky.
56. Thailand, Suphan Buri Province, Dan Change District, Phu Toei National Park, 14°56’N 99°25’E, 360 m a.s.l., H. Malicky.
57. Thailand, Nakhon Pathom Province, Kasetsart University, Kampangsaeng Campus, 14°00’N 99°50’E, 7 m a.s.l., H. Malicky.
58. Thailand, Chon Buri Province, Bang Saen District, 13°25’N 101°03’E, 140 m a.s.l., H. Malicky.
59. Thailand, Chiang Mai Province, Mae Ping River, 18°48’N 98°00’E, 313 m a.s.l., H. Malicky.
60. Thailand, Lamphun Province, Mae Ping River, 18°33’N 98°59’E, 292 m a.s.l., H. Malicky.
61. Thailand, Nonthaburi Province, Chao Phraya River, 13°53’N 100°28’E, 5 m a.s.l., H. Malicky.
62. Thailand, Ayutthaya Province, Chao Phraya River, 14°23’N 100°32’E, 8 m a.s.l., H. Malicky.
63. Thailand, Phitsanulok Province, Chattrakan Waterfall National Park, 17°17’N 100°40’E, 280 m a.s.l., H. Malicky.
64. Thailand, Kanchanaburi Province, Thong Pha Phum District, Ban Anongraksa, 14°39’N 98°35’E, 180 m a.s.l., H. Malicky.
65. Thailand, Surin Province, Sangkha District, Tap Tan River, 14°39’N 103°46’E, 280 m a.s.l., H. Malicky.
66. Vietnam, Dong Nai Province, Nam Cat Tien, 11°42’N 107°43’E, 160 m a.s.l., H. Malicky.
67. Thailand, Nan Province, Mae Charim National Park, 18°36’N 100°58’E, 340 m a.s.l., H. Malicky.
68. Thailand, Chiang Mai Province, Ban Ping Kong, 119°27’N 99°00’E, 410 m a.s.l., H. Malicky.
69. Thailand, Chiang Mai Province, Chiang Dao District, Mae Ping River, 19°23’N 98°01’E, 400 m a.s.l., H. Malicky.
70. Thailand, Chiang Mai Province, Chiang Dao District, Elephant Training Center Chiang Dao, Mae Ping River, 19°23’N 98°01’E, 380 m a.s.l., H. Malicky.
71. Thailand, Chiang Mai Province,Chiang Dao District, Mae Ping River, 19°15’N 98°59’E, 380 m a.s.l., H. Malicky.
72. Thailand, Chiang Rai Province, Mae Sai River, 20°26’N 99°53’E, 400 m a.s.l., H. Malicky.
73. Thailand, Nakhon Nayok Province, Wang Takrai Park, 14°19’N 101°18’E, 60 m a.s.l., H. Malicky.
74. Thailand, Phetchabun Province, Thung Salaeng Luang National Park, 16°56’N 100°53’E, 700 m a.s.l., H.Malicky.
75. Cambodia, Pursat Province, Pursat River, 12°16’N 103°01’E, 300 m a.s.l., H. Malicky.
76. Laos, Luang Prabang Province, Khan River, 19°53’N 102°09’E, 300 m a.s.l., H. Malicky.

The study sites number 1 and 2 are in Mekong Delta; study sites number 3–16 are in the Tonle Sap Basin; study sites number 17–31 and 48–50 are in the Khorat Plateau; study sites number 32–47 are in the Northern Highlands.

For the newly-discovered species of *Ecnomus*, the male genitalia were illustrated using a compound microscopy with a drawing tube, first with pencil and then with Adobe Illustrator. Teminology for genitalic structures follows that of Cartwright (1990). The holotype and paratypes are stored in 70% ethanol and are deposited at Princess Maha Chakri Sirindhorn Natural History Museum, Prince of Songkla University, Hat Yai Campus, Hat Yai District, Songkhla Province, Thailand (PSUNHM). Some paratypes are deposited in the collections of Dr Hans Malicky (CHM) and the Clemson University Arthropod Collection (CUAC).

## Data resources

**Faunistic data on *Ecnomus* species along the Lower Mekong River**

**Figure 2.**
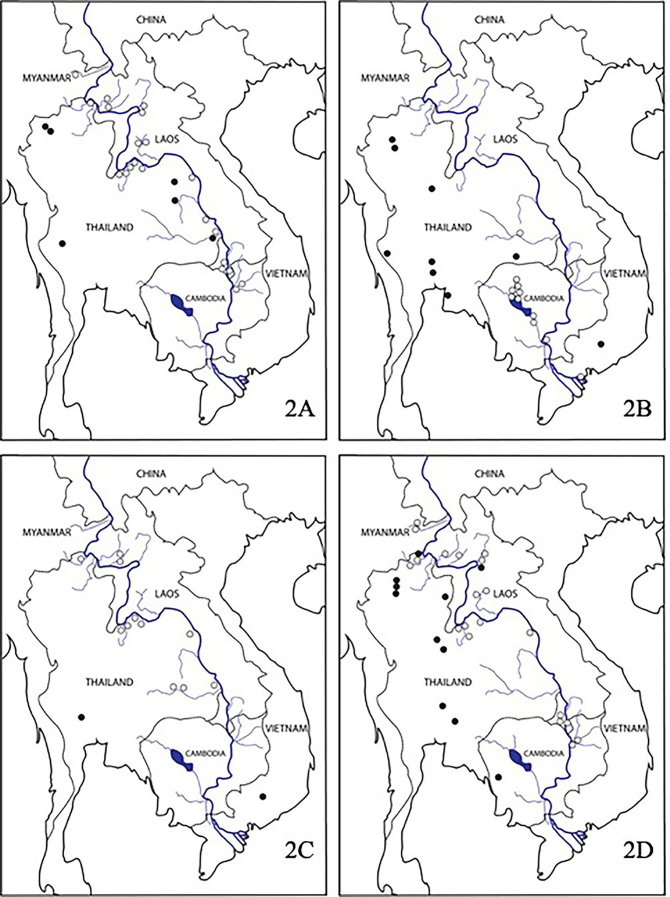
Distribution maps of four common *Ecnomus* spp. from the Lower Mekong River and its tributaries and from Thailand and adjacent regions **2A** *Ecnomusalkmene* Malicky & Chantaramongkol 1997; **2B** *E.mammus* Malicky & Chantaramongkol 1993; **2C** *E.quordaio* Malicky 1993; **2D** *E.volovicus* Malicky & Chantaramongkol 1993. Grey circles = study sites of P. Laudee and P. Thamsenanupap; black circles = study sites of H. Malicky. Outline of the map was redrawn from www.stimson.org.

Twenty-three species of *Ecnomus* spp., including a new species from a tributary of the Lower Mekong River, were collected and identified (Table 1). The most abundant species of *Ecnomus* in this area were *E.alkmene* Malicky & Chantaramongkol, 1997; *E.mammus* Malicky & Chantaramongkol, 1993; *E.quordaio* Malicky, 1993; and *E.volovicus* Malicky & Chantaramongkol, 1993. The distributions of those abundant species were mapped also with data of the species collected by Dr Hans Malicky from adjacent countries. In addition, *E.alkaios* Malicky & Chantaramongkol, 1997 and *E.vibenus* Malicky & Chantaramongkol, 1993 were rare in the areas of study. *Ecnomusalkmene*, *E.volovicus* and *E.quordaio* were dominant species and distributed only in the Northern Highlands and the Khorat Plateau. In contrast, *E.mammus*, *E.bou* Malicky & Chantaramongkol, 1993 and *E.vibenus* were collected mainly from the Tonle Sap Basin. However, *E.ilos* Malicky & Prommi, 2004 is a species that can be found across the Khorat Plateau and Tonle Sap Basin. Moreover, in the area of the Mekong Delta, the numbers of species and number of individuals caught were very low, with only two species identified, *E.mammus* and *E.plotin* Malicky & Laudee, 2010 (Fig. 2).

In this survey, most of the study sites are potamon (river). The species of *Ecnomus*, such as *E.alkmene*, *E.quordaio* and *E.plotin*, were collected only from the main river. However, study sites numbered 7, 8, 9 and 10 are at Tonle Sap Lake; *E.mammus* was collected from those study sites, which means that at least that species can live in lentic habitats. In addition, the study sites numbered 14, 26 and 48 are waterfalls and streams; *Ecnomusjojachin* Malicky & Chantaramongkol 1993 and *E.totiio* Malicky & Chantaramongkol, 1993 were found only in such biotopes (Table 1).

## Taxon treatments

### Ecnomus stungtrengensis

Laudee & Malicky sp. n.

F49255FC-AFC7-5790-8C78-A962ACF19610

5DF269D3-6C08-46B4-855D-2F0BF4B04B50

*Ecnomus* McLachlan, 1864
Type species: *Ecnomustenellus* (Rambur, 1842) (monobasic)

#### Materials

- **Type status:** Holotype. **Occurrence:** sex: male; lifeStage: Adult; occurrenceStatus: present; preparations: whole animal; occurrenceID: 867C6D9C-CF80-52DF-A6A2-C0B38E0A1A4C; **Taxon:** scientificName: *Ecnomusstungtrengensis*; nameAccordingTo: Province; kingdom: Animalia; phylum: Arthropoda; class: Insecta; order: Trichoptera; family: Ecnomidae; genus: Ecnomus; specificEpithet: *stungtrengensis*; scientificNameAuthorship: Laudee and Malicky; **Location:** continent: Asia; country: Cambodia; countryCode: KH; stateProvince: Krung Stung Treng; county: Cambodia; municipality: Krung Stung Treng; locality: Tonle Sekong River, 13°32’00”N, 105°59’56”E; minimumElevationInMeters: 42; **Identification:** identifiedBy: Laudee and Malicky; **Event:** samplingProtocol: UV light trap; samplingEffort: 1 trap-night; year: 2019; month: 4; day: 8; **Record Level:** type: Trichoptera; language: English; institutionCode: PSUNHM; basisOfRecord: PreservedSpecimen
- **Type status:** Paratype. **Occurrence:** sex: male; lifeStage: Adult; occurrenceStatus: present; preparations: whole animal; occurrenceID: FCDEB7C7-94E0-50B2-BC0A-8E6B9AB20351; **Taxon:** scientificName: *Ecnomusstungtrengensis*; nameAccordingTo: Province; kingdom: Animalia; phylum: Arthropoda; class: Insecta; order: Trichoptera; family: Ecnomidae; genus: Ecnomus; specificEpithet: *stungtrengensis*; scientificNameAuthorship: Laudee and Malicky; **Location:** continent: Asia; country: Cambodia; countryCode: KH; stateProvince: Krung Stung Treng; county: Cambodia; municipality: Krung Stung Treng; locality: Tonle Sekong River, 13°32’00”N, 105°59’56”E; minimumElevationInMeters: 42; **Identification:** identifiedBy: Laudee and Malicky; **Event:** samplingProtocol: UV light trap; samplingEffort: 1 trap-night; year: 2019; month: 4; day: 8; **Record Level:** type: Trichoptera; language: English; institutionCode: 2 males (PSUNHM), 2 males (CHM), 2 males (CUAC); basisOfRecord: PreservedSpecimen

#### Description

**Adult habitus**: Specimens in alcohol with head, thorax, forewings, abdomen and legs light brown.

**Figure 3.**
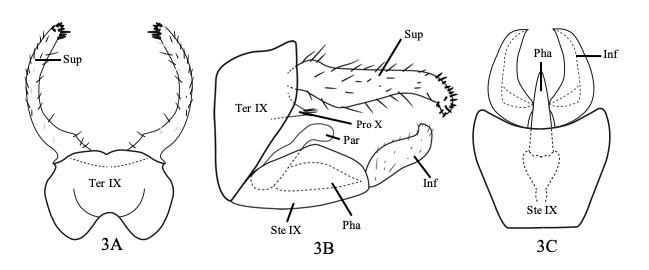
*Ecnomusstungtrengensis*, **sp. n.** Male genitalia. **A** tergum IX and superior appendages, dorsal; **B** tergum IX, sternum IX, superior appendages and inferior appendages, left lateral; **C** sternum IX and inferior appendages, ventral. Ter IX = tergum IX, Ste IX = sternum IX, Sup = superior appendage (paired), Inf = inferior appendage (paired), Pro X = process of segment X, Par = paramere (paired), Pha = phallus.

**Male** (Fig. 3) Length of each male forewing 4.0 mm (3.5–4.5, n = 5); Tergum IX somewhat rounded, anterior margin bilobed, posterior margin slightly incised in dorsal view (Fig. 3A); trapezoidal, nearly triangular and truncate anteriorly in lateral view (Fig. 3B). Sternum IX ovoid in lateral view (Fig. 3B); isosceles trapezoid in ventral view and evenly concave posteriorly (Fig. 3C); Superior appendages long, slender, strongly and evenly curved inwards posteriorly with numerous long setae, spiny setae subapically in dorsal view (Fig. 3A); in lateral view, superior appendages, long, slender, tapered and bent downwards with numerous spiny setae apically (Fig. 3B). Process of segment X triangular with strong apical setae in lateral view. Inferior appendages cylindrical with numerous small setae, bent slightly inwards and upwards subapically, rounded apically in lateral view (Fig. 3B); in ventral view, crescent-shaped, expanded basally with inner submedial lobe, rounded apicolaterally, acute apicomesally (Fig. 3C). Paramere spoon-like, bent downwards subapically, rounded apically. Phallus shaped like candle flame, pointed apically in lateral view (Fig. 3B); in ventral view, conical with pointed apex (Fig. 3C).

#### Diagnosis

The male genitalia of *E.stungtrengensis* sp. n. are similar to those of *E.robustior* Ulmer, 1951, *E.pseudotenellus* Ulmer, 1930 and *E.projectus* Li & Morse, 1997, but clearly different from those species by the shape of the superior appendages. In the new species, they are slender and bent downwards distally in lateral view, but in the other three species, they are straight and not bent in lateral view. In dorsal view, the superior appendages of the new species are bent evenly mesad to define 3/4 of a circle, but in the other three species, they are straight.

#### Etymology

The species is named for the type locality, Krung Stung Treng Province, with the epithet having masculine gender, corresponding with the gender of the genus.

#### Distribution

Cambodia (Krung Stung Treng Province).

## Discussion

This survey provides new species records for genus *Ecnomus* in Cambodia including *E.alkmeme* Malicky & Chantaramongkol, 1997; *E.atevalus*; *E.bou*; *E.digitatus* Mosely, 1932; *E.dromiel*; *E.ilos*; *E.jojachin*; *E.mammus*; *E.obtusus*; *E.paget*; *E.pseudotenellus*; *E.totiio* and and *E.vibenus*. In addition, *E.digitatus*, *E.dromiel*, *E.quordaio*, *E.totiio* and *E.triangularis* Sun, 1997 are new species records for Laos. Additionally, *E.plotin* is a new species record for Vietnam. Moreover, *E.obtusus* and *E.triangularis* are new species records for Thailand (Armitage et al. 2005, Malicky 2010a, Laudee and Malicky 2017).

*Ecnomusplotin* was collected only in the Mekong Delta area. This species was described from Thailand where the habitat is a river mouth (Malicky 2010b). We expected that *E.plotin* is a brackish-water species of caddisfly which lives in river mouths where the water is somewhat saline. Several Trichoptera larvae are known to tolerate or prefer brackish water of low salt concentration (e.g. *Oxyethirasimplex* Ris, 1897, *Limnephilusgraecus* Schmid, 1965, *Limnephilusminos* Malicky, 1971 and *Triaenodesochreellus* McLachlan, 1877) or may survive in high salt concentrations, such as *Limnephilusaffinis* Curtis, 1834 (Sutcliffe 1961, Malicky 1974, Malicky 2014) The four-known species of the family Chathamidae live only in seawater (Neboiss 1961, Leader 1971).

Most of the study sites are potamon (rivers), except that study sites numbered 7, 8, 9 and 10 are Tonle Sap Lake; *Ecnomusmammus* was collected from those study sites which means that at least some *Ecnomus* species can live in lentic habitats. Wells and Yule (2008) reported that *Ecnomusselangor* Wells and Yule, 2008 lives in a peat swamp, another kind of standing water habitat. The study sites numbered 14, 26 and 48 are waterfalls and streams. *Ecnomusjojachin* and *Ecnomustotiio* were found only in such biotopes. Laudee and Malicky (2014) reported that *Ecnomustotiio* occurred in waterfalls in southern Thailand. In addition, Laudee et al. (2020) reported two new species of *Ecnomus* spp. from a waterfall in southern Laos. This survey showed that species of the genus *Ecnomus* can occupy a variety of aquatic biotopes, including both lentic and lotic habitats. Moreover, many species of *Ecnomus* are potamon species which live in rivers, but also some species are rhithral species occupying small streams and waterfalls. General environmental characteristics at a collection site might not always represent those environmental features that are critical to the presence of a species; some species may require specific microhabitat that is not the dominant microhabitat and was not sampled or recorded by the collectors.

## Supplementary Material

XML Treatment for Ecnomus stungtrengensis

## Figures and Tables

**Table 1.** Diversity of *Ecnomus* spp. along the Lower Mekong River and its tributaries, showing study site numbers corresponding with those in Fig. 1 (and number of male specimens in parentheses).

Taxa	Study sites
*Ecnomusalkmene* Malicky & Chantaramongkol, 1997	15(6), 16(8), 17(50),
	18(100+), 19(48), 20(100+),
	21(6), 25(19), 27(100+),
	28(3), 29(23), 30(100+),
	31(10), 38(2), 40(3), 41(1),
	42(1), 46(22), 47(3), 49(3),
	50(1)
*Ecnomusalkaios* Malicky & Chantaramongkol, 1997	37(1)
*Ecnomusatevalus* Malicky & Chantaramongkol, 1993	11(1), 15(3), 24(13), 35(1),
	36(1)
*Ecnomusbou* Malicky & Chantaramongkol, 1993	12(10)
*Ecnomuscaesar* Malicky & Chaibu, 2000	50(1)
*Ecnomuscincibilus* Malicky & Chantaramongkol, 1997	26(1), 35(1)
*Ecnomusdigitatus* Mosely, 1932	12(3), 50(2)
*Ecnomusdromiel* Malicky, 2014	15(4), 17(18), 18(18), 19(6),
	43(3)
*Ecnomusilos* Malicky & Prommi, 2004	3(3), 4(1), 5(8), 6(4), 22(1),
	23(1), 25(8)
*Ecnomusjojachin* Malicky & Chantaramongkol, 1993	26(1), 14(2)
*Ecnomusmammus* Malicky & Chantaramongkol, 1993	2(1), 3(2), 5(1), 6(4), 7(1), 8(100+),
	9(73), 10(7), 11(3), 12(4), 22(1)
*Ecnomusobtusus* Ulmer, 1910	13(1), 34(4)
*Ecnomuspaget* Malicky & Chantaramongkol, 1997	15(3), 20(10), 31(1)
*Ecnomusplotin* Malicky & Laudee, 2010	1(6)
*Ecnomuspseudotenellus* Ulmer, 1930	15(10), 25(9), 29(1), 30(3)
*Ecnomuspuro* Malicky & Chantaramongkol, 1993	20(1), 22(31), 23(8), 25(12),
	27(3), 28(3), 30(6), 31(1),
	37(3), 43(1), 44(4)
*Ecnomusrobustior* Ulmer, 1951	32(3), 33(14)
*Ecnomusstungtrengensis* sp. n.	15(6)
*Ecnomustotiio* Malicky & Chantaramongkol, 1993	14(26), 48(11)
*Ecnomustriangularis* Sun, 1997	27(1), 31(1), 41(1)
*Ecnomusvibenus* Malicky & Chantaramongkol, 1993	12(1)
*Ecnomusvolovicus* Malicky & Chantaramongkol, 1993	15(5), 16(2), 17(18), 18(48), 19(12),
	25(1), 27(100+), 30(100+), 31(10),
	32(1), 37(1), 39(1), 40(3), 41(5),
	44(100+), 45(1), 46(6), 48(7), 49(2)
